# An improved camera trap for amphibians, reptiles, small mammals, and large invertebrates

**DOI:** 10.1371/journal.pone.0185026

**Published:** 2017-10-05

**Authors:** Michael T. Hobbs, Cheryl S. Brehme

**Affiliations:** 1 Wildlife Ecologist, Technologist, San Jose, California, United States of America; 2 Western Ecological Research Center, U.S. Geological Survey, San Diego, United States of America; University of Sydney, AUSTRALIA

## Abstract

Camera traps are valuable sampling tools commonly used to inventory and monitor wildlife communities but are challenged to reliably sample small animals. We introduce a novel active camera trap system enabling the reliable and efficient use of wildlife cameras for sampling small animals, particularly reptiles, amphibians, small mammals and large invertebrates. It surpasses the detection ability of commonly used passive infrared (PIR) cameras for this application and eliminates problems such as high rates of false triggers and high variability in detection rates among cameras and study locations. Our system, which employs a HALT trigger, is capable of coupling to digital PIR cameras and is designed for detecting small animals traversing small tunnels, narrow trails, small clearings and along walls or drift fencing.

## Introduction

Camera traps are valuable sampling tools commonly used by ecologists and conservationists to inventory and monitor wildlife communities [1–6], estimate occupancy and abundance [1, 3, 4], and monitor animal behavior [1, 7], especially for rare, threatened, and endangered species [2, 8, 9]. Most digital game and trail cameras use a passive infrared (PIR) sensor for their trigger in order to capture images. The PIR sensor is a pyroelectric device designed to detect mammals based on a combination of heat and motion [9–13]. The PIR sensor responds to thermal emissions (radiation) within wavelengths ranging from 8 μm to 14 μm, which is the average range an endothermic mammal radiates [10, 11, 14]. It is the comparative change in infrared emissions between an object and its background, differentiated between thermally sensitive crystals inside the PIR sensor that triggers detection [11]. The majority of these cameras also allow researchers to adjust operational parameters (i.e. trigger sensitivity, photo quantity, delay between pictures, time-lapse, etc.) and capture metadata such as date, time and temperature [9, 12].

Typically an animal must be 2.7°C warmer than its surrounding environment, and moving across a PIR sensor’s field of view, to trigger a detection [9, 15]. However, ectothermic animals rarely vary greater than 3°C from their surrounding environment [16, 17]. The distance of an animal from the camera’s PIR sensor, as well as its body mass, also determines the magnitude of infrared radiation, which affects the ability of the PIR to trigger an image [10, 13, 18]. Therefore a larger animal having greater mass generates a stronger heat signature than a smaller animal having less mass [2, 5], or one further away [10]. Because of these limitations, PIR triggers perform well for large and medium sized mammals [2, 9, 18], but do not reliably detect ectotherms (reptiles, amphibians, and invertebrates) or smaller mammals [2, 3, 19–21].

Consequently, live-trapping and other time intensive methods are still the standard for studying small animals [22–29]. However, these methods (Sherman live traps, funnel trap and pitfall traps) are labor intensive, costly and if not managed properly can cause animals stress or mortality from prolonged retention or exposure [22, 24, 27, 30], predation [22, 24, 28, 29], drowning [28], starvation [28], or aggression among multiple captured animals [29, 24]. Therefore, passive and reliable sampling methods for research and monitoring small animals are desirable.

Researchers have used camera traps to study small animals with varying success [19, 21, 30]. Increasing the PIR sensitivity of the camera is used to increase the probability of detecting small animals [7, 9, 19, 30]. However, this often results in a high ratio of false triggers [9]. Though the PIR sensor may be acting as designed, a multitude of thermal changes in the environment, such as moving vegetation [9], thermal air masses [9, 18], and uncontrolled electrical noise can trigger the camera [10]. Even in relatively small studies using high sensitivity PIR settings, false triggers can result in hundreds or thousands of images that are cost and time prohibitive to sort through [4, 7]. These false triggers decrease battery life and cause premature filling of memory cards [19], especially when capturing video.

While detection can vary widely among cameras, there are often significant differences in sensitivity among individual cameras of the same make and model [9, 12]. This can be attributed to different environmental conditions at sampling locations and slight changes in camera orientation, which are difficult or impossible to control when surveying multiple sites. Even when perfectly controlled, Hughson et al. [12] found up to a 100% difference in the photographic rates of identical cameras with the same orientation and focus. This camera-to-camera variability is a problem for most studies, but is even more pronounced when challenging the limits of detection. Thus, the use of PIR detection for studies of small animals may result not only in high costs related to false triggers but also to inaccurate study conclusions, particularly if detection probability is confounded with sampling location.

To remove problems with camera variability and false triggers, others have employed time-lapse triggering to study small animals, particularly for studies of reptiles and amphibians that have a very low heat differential with the surrounding environment [19, 20]. Time-lapse is advantageous in that it allows for a controlled level of sampling effort across cameras, sites and time. However, this also results in a multitude of blank images that are cost-and time-prohibitive to sort through, and suffers from low detection rates [4, 19]. For instance, a time-lapse frequency of one-minute per picture would create 1,440 images over 24 hours for each camera and still sample less than 2% of the total time monitored. In a two-year tunnel crossing study of long-toed salamanders (*Ambystoma macrodactylum*) [19] use of timed-lapse triggering produced over one million images capturing fewer than 2000 animal images.

Finally, some attempts have been made to reduce the time and effort in sorting out false trigger images using computer software with imaging filters that can weigh variations in pixilation against its background, thereby identifying pictures void of target animals, or identifying target species with some success [7]. However, even this approach is often challenged when environmental factors such as vegetation movement, or shifting shadows from cloud cover and changing light conditions outweigh pixel variations within the background [7].

**Fig 1:**
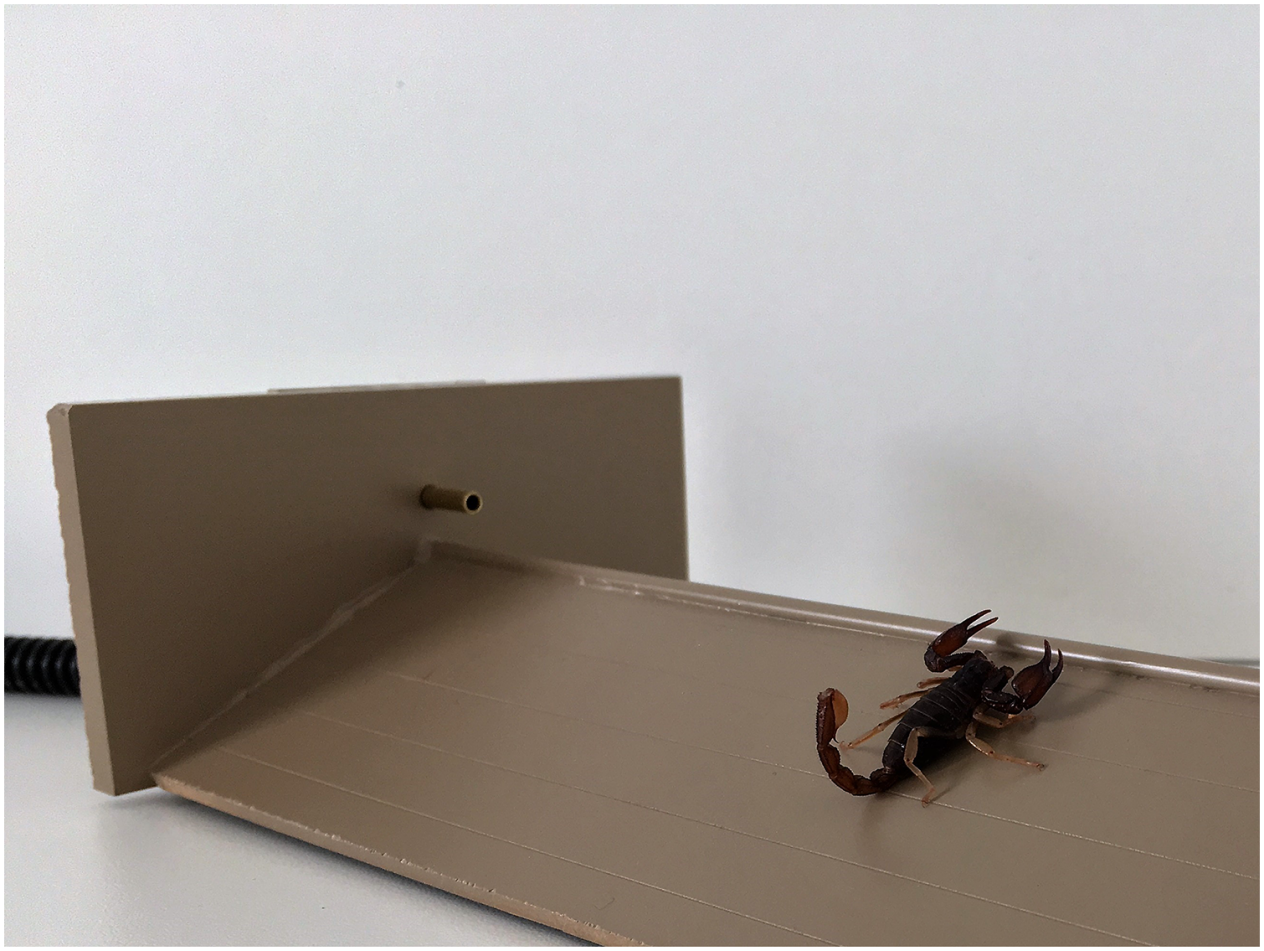
Elevated threshold with pre-aligned optical beam running parallel to crest. Slant of threshold facilitates deflection of leaves and other detritus.

It has been suggested that active camera traps need to be re-evaluated to assist researchers in the science community [9]. We describe an active optical trigger that targets small animals and alleviates many of the challenges posed by PIR triggers. The Hobbs Active Light Trigger (HALT) is an optical trigger similar to the TrailMaster 1500 and 1550 [1, 31–33]. The difference is in scope and technology. The HALT is a 3 mm pre-aligned near infrared (NIR) beam mounted above and parallel to an elevated threshold targeting small ectothermic animals (patent pending) (Fig 1). The elevated threshold can vary in height and length, not exceeding 36 cm in length and is designed to divert falling vegetation and protect the beam. The termination points of the beam (both transmit and receive) are extended 1 cm by using a 3 mm tube. The tube reduces the amount of unwanted triggers created by small invertebrates walking across either termination point. In comparison, the TrailMaster is manually aligned with a 1 cm infrared (IR) beam, ranging up to 30 m. The beam is elevated to an animal’s shoulder over open terrain, primarily targeting larger mammals. The HALT can currently be coupled to many game and trail cameras offering additional advantages.

In this study, we compared the HALT trigger to current PIR trigger technologies by modifying the circuits of two brand name PIR camera traps. Using two unmodified PIR camera traps as controls, we assessed differences in the probability of detecting objects of varying size, temperature and speed. We also tested the ability of the HALT to detect small animals under field conditions.

## Materials and methods

### Controlled study

To compare PIR camera traps to our new HALT system, we purchased two Bushnell cameras (B1, B2) and two Reconyx cameras (R1, R2). The Reconyx cameras were customized for high PIR sensitivity at the factory. For PIR triggered detection, one each of Bushnell and Reconyx were unmodified (B2, R2) and used as controls. The control cameras were set to high sensitivity with one picture per trigger. For HALT detection, one each of Bushnell and Reconyx (B1, R1) were modified and used as the experimental treatment. Each camera used SanDisk 8 GB class 10 SD memory cards and 12 Duracell AA batteries.

**Fig 2:**
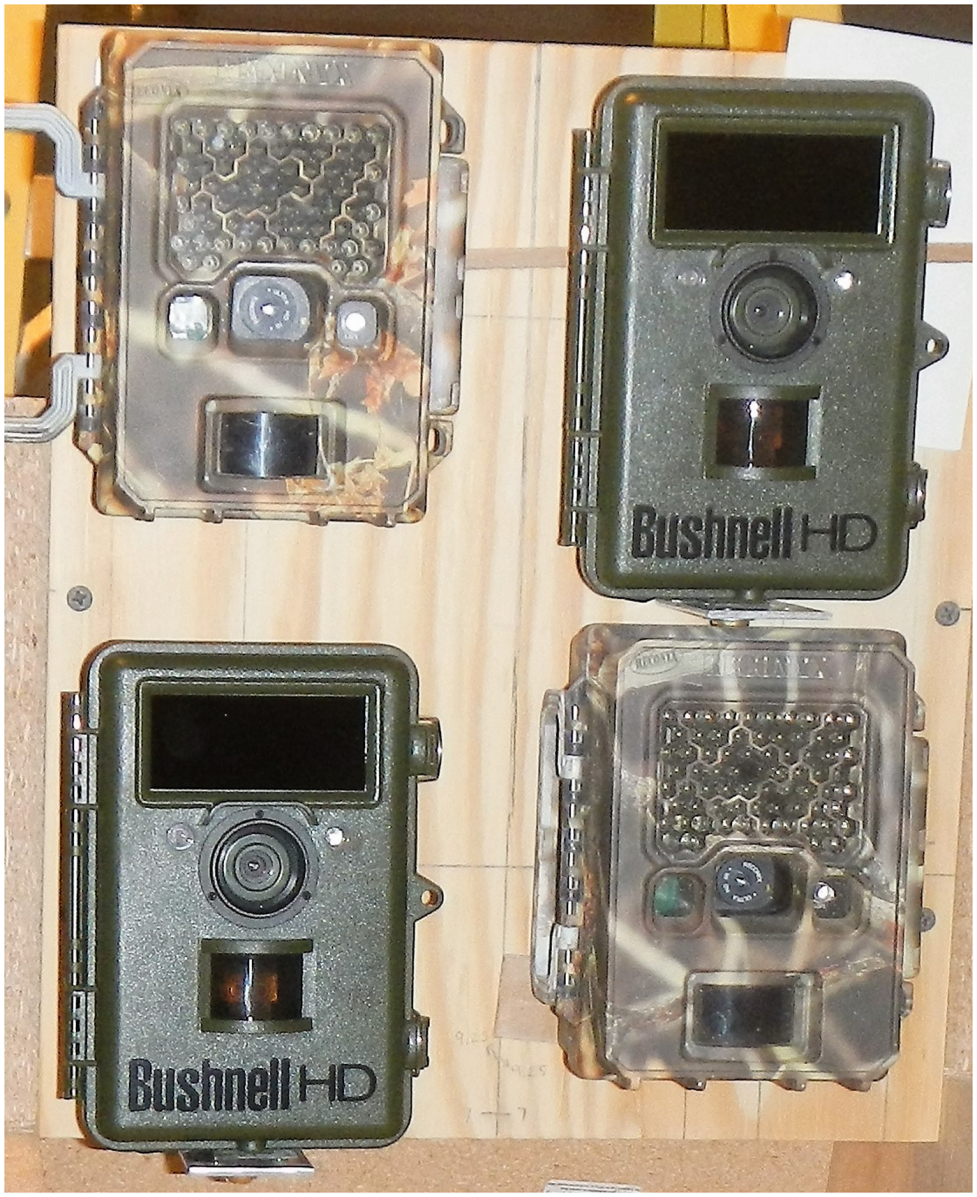
Experimental and Control cameras (B1, R1, B2, R2) aligned to center of backdrop.

**Fig 3:**
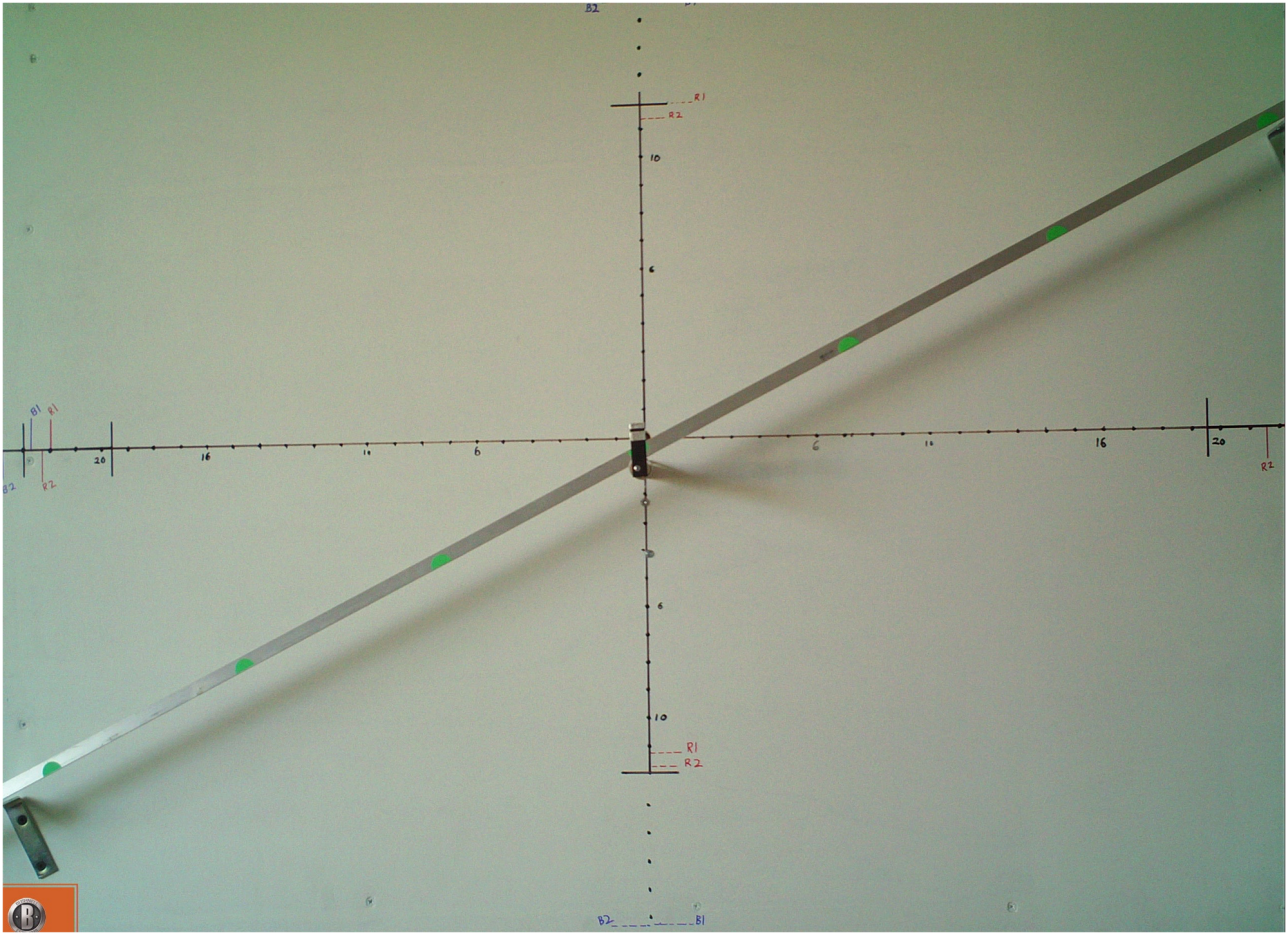
Backdrop field of view (FOV) with 45° guide rail.

All four cameras (B1, B2, R1, R2) were mounted on a single board, 30 cm wide by 36 cm high, stacked two on top of two (Fig 2). A backdrop board (122 cm wide by 91 cm high) was mounted 1.5 m away from camera lenses encompassing each camera’s field of view (FOV) (Fig 3). The distance from camera lens to backdrop was 1.5 m. Depending on camera type, the FOV measured at the backdrop was 58 cm wide by 112 cm high for (R1, R2) and 84 cm wide by 117 cm high for (B1, B2). A track rail was mounted at a 45° angle along the backdrop to accommodate targets moving across each camera’s FOV consistently and accurately. A single optical trigger was shared between cameras B1 and R1 and mounted to the rail at the center of the backdrop. All cameras’ FOV were aligned to the center of the backdrop.

Under controlled conditions, we tested the effects of body size, body temperature and speed on the probability of detecting moving targets. Target size, temperature and speed were varied to represent an array of small wildlife species and field conditions. Three different size targets were made from hardwood ovals and balls covered with faux fur in the categories of extra small (XS/4.7 cm x 4.0 cm/21 g), small (SM/9.0 cm x 4.5 cm/50 g) and medium (MED/16.0 cm x 6.5 cm/184 g).

While maintaining an ambient temperature of 21°C, each target was tested at three different temperatures: 21°C, 24°C and 35°C (0°, +3° and +14°). Neutral (0°) and 3°C above ambient conditions represented the expected temperature differential for ectothermic animals (invertebrates and herpetofauna) as well as endothermic animals (small mammals) in hot conditions [9, 17]. The 14°C above ambient temperature level represented the larger temperature differential between small mammals and ambient conditions under many field situations. The temperature of each target was modified and controlled by submerging and encasing targets in thermally controlled sand. Each target was measured before and after each run by a digital infrared thermometer to ensure temperature control.

Finally, we tested each target at each temperature at three speeds; slow (1 cm/sec), medium (20 cm/sec) and fast (1 m/sec) to represent the variation in natural movement speeds of invertebrates, herpetofauna and small mammals. Slow target speeds (1 cm/sec) were achieved by using a dowel marked in centimeters to push each target up the 45° track rail while pacing to a one second metronome. Medium speeds (20 cm/sec) were achieved by using a string marked in 20cm increments allowing target to descend (gravity feed) down the 45° track rail while pacing to a one second metronome. Fast target speeds (1 m/sec) were achieved by allowing targets to free-fall (gravity feed) from top of 45° track rail to bottom. A preventive shield (cardboard) was placed on either side of the backdrop board to mitigate incidental take of images during each experiment.

For each experimental condition—size, temperature, and speed—we ran six replicate runs for a total of 162 runs. For each run, we recorded if the PIR or HALT triggered a photo for each camera. We considered it a positive detection if the target was captured in the corresponding photo while referencing time stamps. Time stamp references determined positive detection from any false triggers.

We estimated detection probabilities of the PIR trigger versus HALT trigger for all experimental conditions using the log-linear modeling program, PRESENCE2 [34]. The occupancy parameter was fixed at one to represent that we knew the target was present under all conditions and trials. We compared models with all combinations of the covariates (size, temperature, and speed). We used Akaike’s Information Criterion (AIC) and model selection procedures described by [35] to rank and compare models.

### Field trials

**Fig 4:**
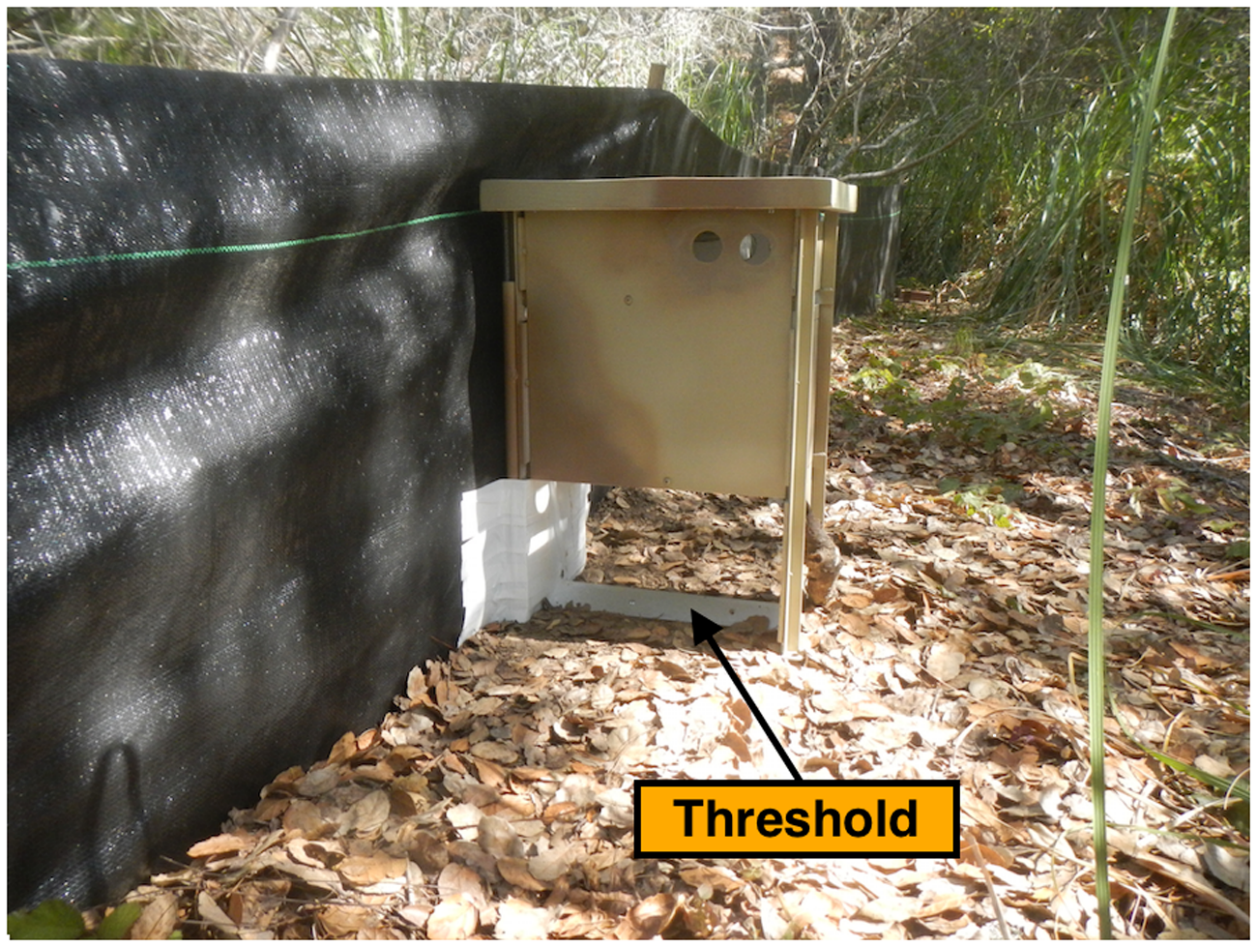
Drift fence with camera trap box. Camera inside locked box with battery and mounted vertically above HALT threshold.

The first field trial was conducted during seasonal breeding migrations of the Santa Cruz long-toed salamander (*Ambystoma macrodactylum croceum*) (SCLTS) at Buena Vista Pond, (Santa Cruz County, CA). Two camera trap systems were installed and active from October 16, 2015 to February 29, 2016. The modified HALT camera trap systems, composed of Bushnell cameras modified with HALT and an external 5 Ah rechargeable lead-acid battery, were encased in a special built wood frame box and placed in-line and parallel to existing drift fencing (Fig 4). One camera trap was located on the inbound side of the pond, the other on the outbound side.

**Fig 5:**
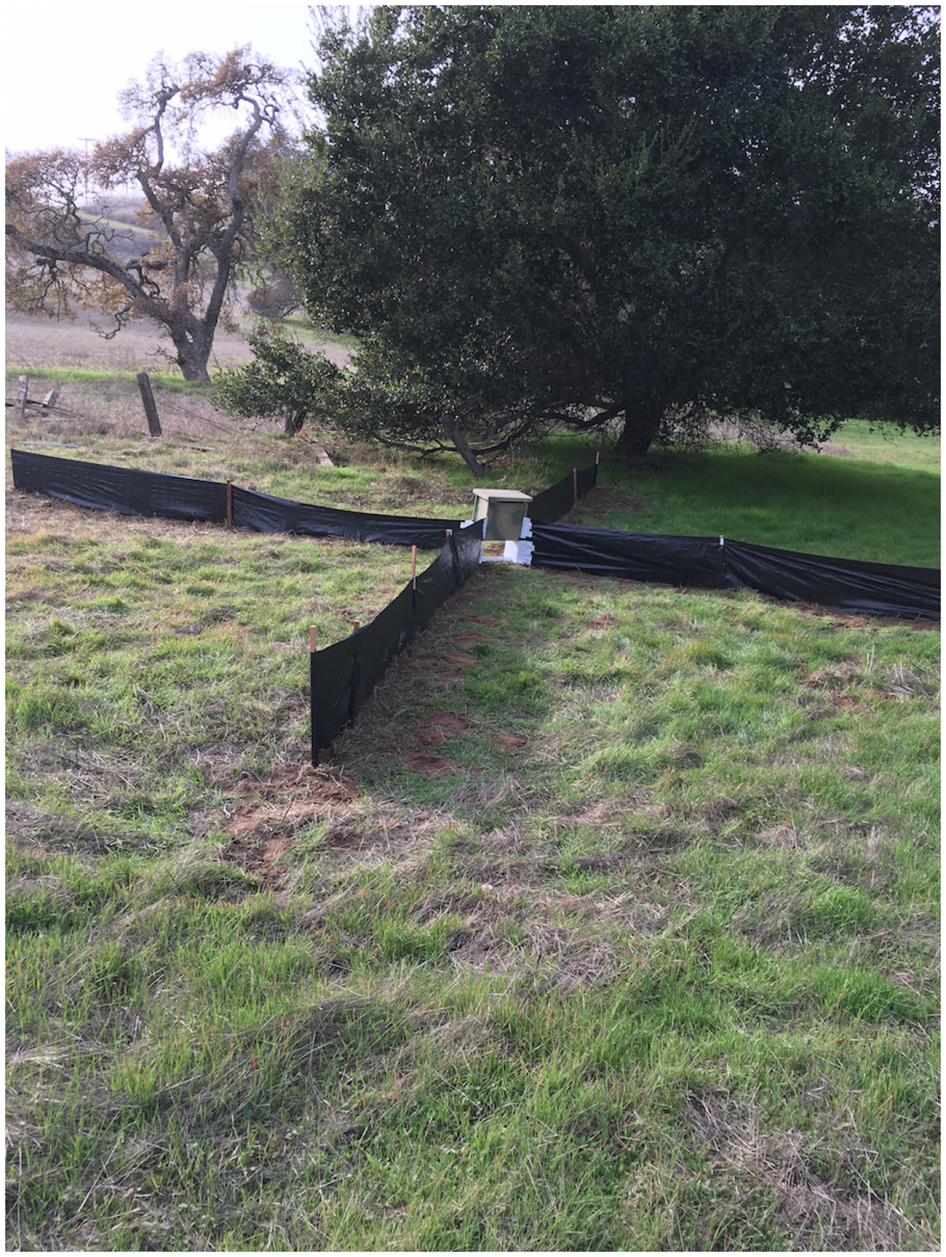
Camera trap drift fence in ‘X’ formation aligned in pathway of suspected CTS corridor.

The second field trial was conducted during seasonal breeding migrations of the California tiger salamander (*Ambystoma californiense*) (CTS) in the foothills at Stanford University (Santa Clara County, CA). Three modified HALT camera trap systems as described were sequentially placed along suspected CTS migration paths between December 07 and December 16 of 2015 and operated until February 22, 2016. Drift fence was installed at the openings of each trap forming an ‘X’ configuration that extended 4.5 m in each direction to funnel animals to the HALT threshold (Fig 5).

## Results

### Controlled study

**Fig 6:**
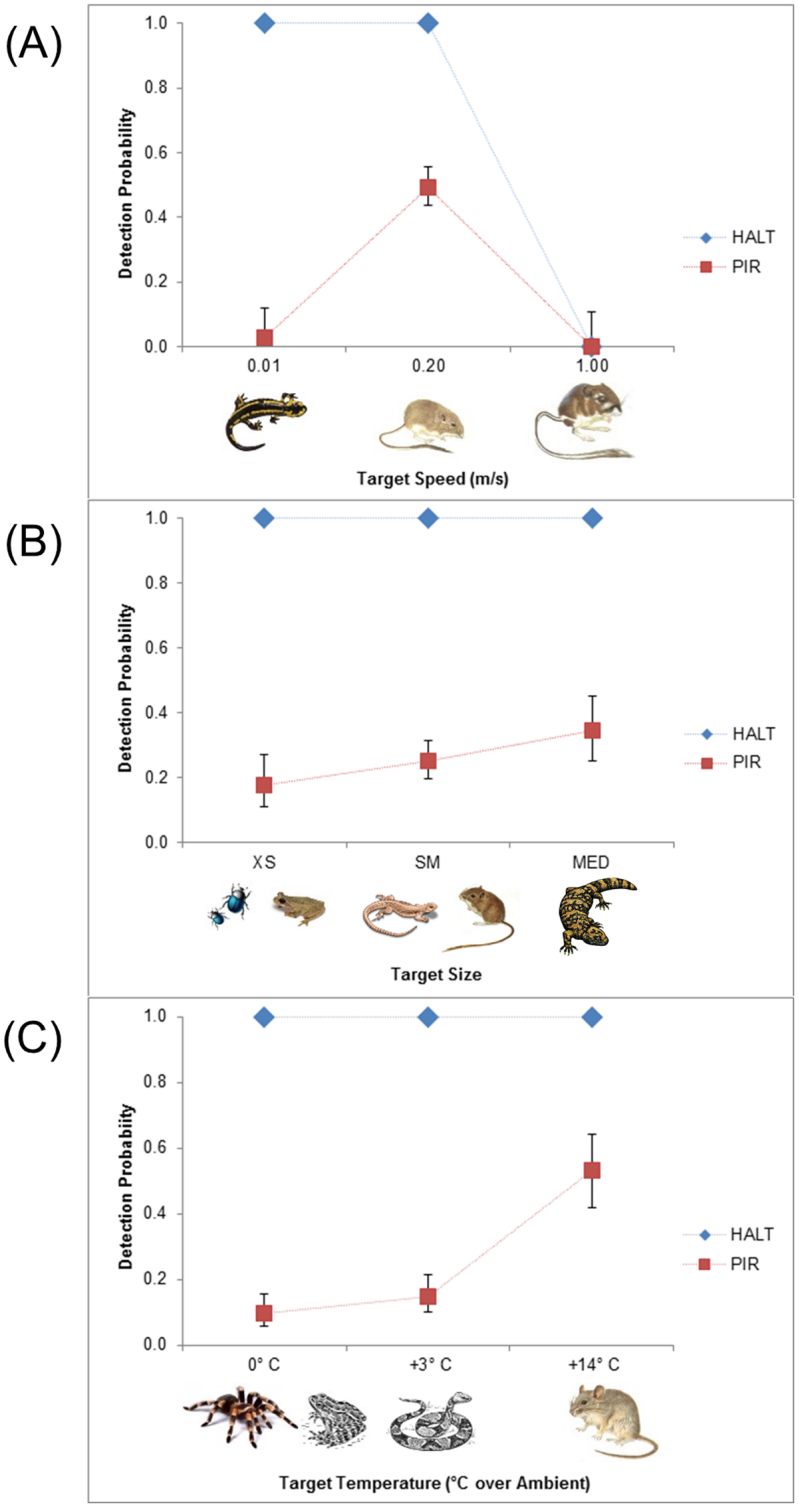
Mean (+/- 1 se) detection probabilities of PIR and HALT modified cameras in relation to target speed (A), target size (B), and temperature (C). Small mammal illustrations reprinted from Mammals of California, Revised Edition by E.W. Jameson Jr. and Hans J. Peeters, edited by Phyllis M. Faber and Bruce M. Pavlik. (c) 2004 by the Regents of the University of California. Published by the University of California Press under a CC BY license, with permission from University of California Press, original copyright 2004.

A total of 272 target images were captured with the four cameras (PIR = 56, HALT = 216). No camera captured images of animals at the fastest movement speed of 1 m/s. These data were excluded from predictive modeling to obtain better fits and avoid overparameterization. In the model comparison, an additive model containing all variables of trigger type, speed, size, and temperature best explained detection probabilities across the study (Table 1). Trigger type had the largest effect on detection probability with an overall estimate of 1.0 (se = 0.0) for HALT in comparison to 0.26 (se = 0.03) for the PIR cameras. Detection probability estimates from the PIR cameras varied with speed, size, and temperature (Fig 6A–6C).

**Table 1:** Comparison of models to predict detection probability across trigger type and target variables.

Model	AIC	Delta AIC	AIC wgt	Model Likelihood	no.Par.	-2*Log Likelihood
p(Trigger +Speed +Temp +Size)	105.49	0	0.66	1.00	6	93.49
p(Trigger +Speed +Temp*Size)	107.09	1.6	0.30	0.45	7	93.09
p(Trigger +Speed*Size*Temp)	110.82	5.33	0.05	0.07	10	90.82
p(Trigger +Speed+ Temp)	117.58	12.09	0.00	0.00	5	107.58
p(Trigger +Speed*Temp)	119.44	13.95	0.00	0.00	6	107.44
p(Trigger +Speed)	185.1	79.61	0.00	0.00	4	177.10
p(Trigger +Temp)	213.73	108.24	0.00	0.00	4	205.73
p(Trigger +Size)	249.97	144.48	0.00	0.00	4	241.97
p(Trigger)	253.23	147.74	0.00	0.00	3	247.23
p(Speed)	550.37	444.88	0.00	0.00	3	544.37
p(Temp)	557.54	452.05	0.00	0.00	3	551.54
p(Size)	573.36	467.87	0.00	0.00	3	567.36
p(.)	573.51	468.02	0.00	0.00	2	569.51

Both PIR and HALT systems were unable to capture images of the high-speed animals (1 m/sec) due to the trigger speed limitations inherent in each camera, relative to the field of view. While the HALT system had perfect probability of detecting animals at the slow and medium speeds of 1 cm/sec and 20 cm/sec (ρ = 1.0, SE = 0.0), the PIR cameras were largely only able to detect animals moving at the medium speed of 20 cm/sec (ρ = 0.49, SE = 0.05; Fig 6A).

The HALT system had a perfect probability of detecting all animal sizes (ρ = 1.0, SE = 0.0). The detection probability of the PIR cameras was low for all sizes in comparison with the HALT, but increased with increasing size of the animal so that the mean probability of detecting the largest target was approximately twice that of the smallest target (XS ρ = 0.18, SE = 0.04; SM ρ = 0.25, SE = 0.03; MED ρ = 0.35, SE = 0.05; Fig 6B).

The HALT system had a perfect probability of detecting animals at all temperatures (ρ = 1.0, SE = 0.0). The mean detection probability of the PIR cameras increased significantly with a greater temperature differential between the animal and ambient temperature (0°C ρ = 0.10, SE = 0.03; 3°C ρ = 0.15, SE = 0.03; 14°C ρ = 0.53, SE = 0.06; Fig 6C).

**Fig 7:**
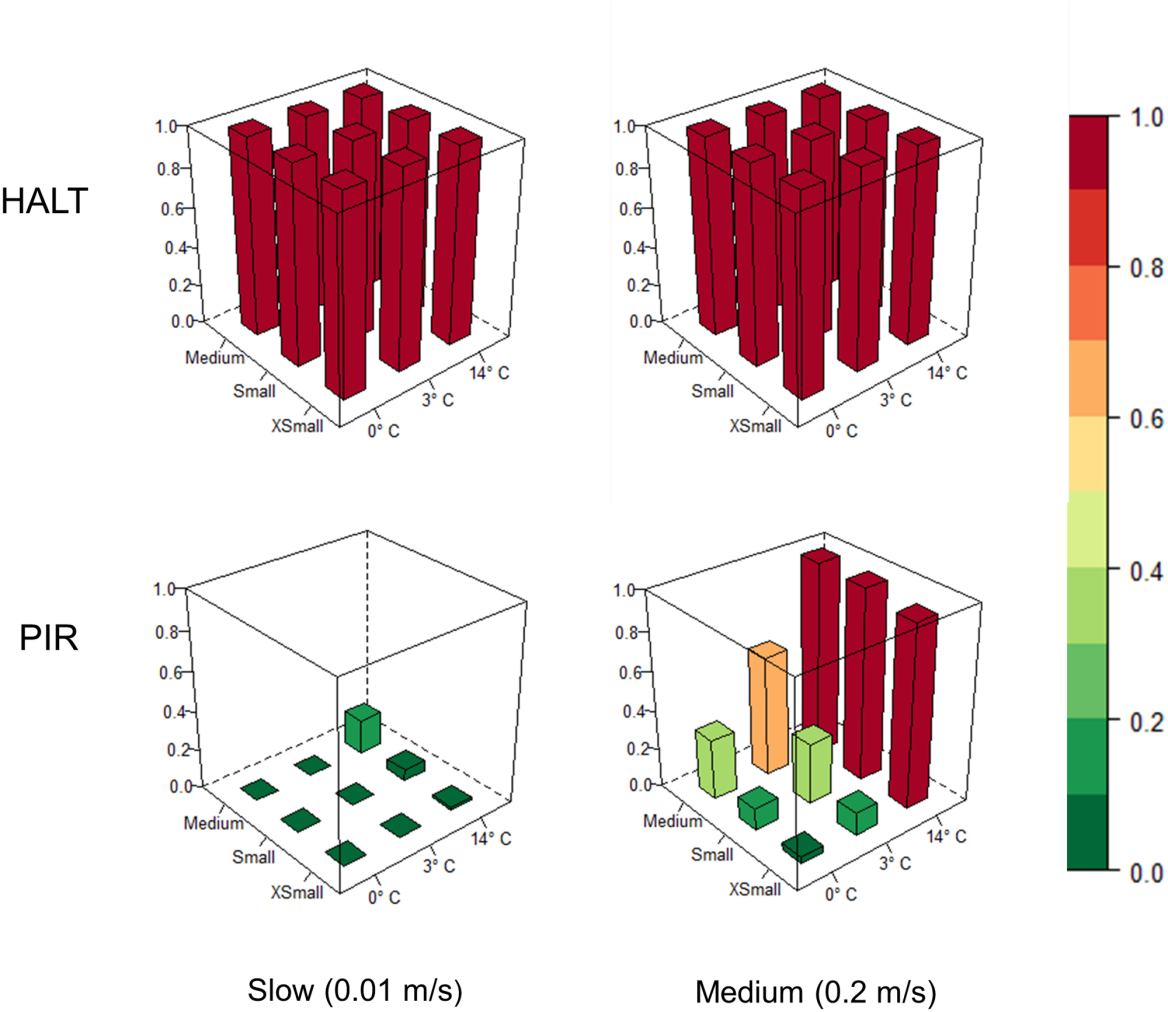
Average individual detection probabilities of PIR cameras in comparison to HALT modified cameras for all combinations of target speed, target size, and temperature.

Individual detection probability estimates showed that PIR cameras were very efficient at capturing images of all size targets at the medium speed (0.2 m/s) and with a temperature differential of 14°C. However, detection probabilities were substantially lower for all other combinations of experimental factors (Fig 7).

### HALT field trials

At Buena Vista Pond, there was a combined total of 252 camera days with a total of 1,236 trigger events. Average images-per-day was five. Of these, 861 (70%) contained animal images and 375 (30%) were blank. As viewed by subsequent video, the blank images likely occurred from fast moving deer mice, fast flying insects and very small invertebrates inhabiting the 3 mm beam extension tubes. Animal images represented a total of five animal groups and 19 species (or genera). This included six species of amphibians, two species of reptiles, four species of mammals, two species of birds, and three genera of invertebrates. Of federally listed salamander species, a total of 76 SCTLS and two CTS passes were detected.

At the Stanford University foothills site, there was a combined total of 220 camera-days with 1,736 trigger events. Average images-per-day was eight. Of these, 1074 (62%) contained animal images and 662 (38%) were blank. As viewed by subsequent video, blank images were caused by mud splashes during several heavy rain events, very small invertebrates crawling inside the 3 mm beam extension tubes and deer mice running across the beam. Positive images represented a total of five animal groups and 14 species (or genera). This included four species of amphibians, three species of mammals, four genera of invertebrates and one bird species. Of federally listed salamander species, a total of four CTS passes were detected.

An image catalog composition of species (or genera) captured at Buena Vista and Stanford can be seen in supporting information (S1 Appendix).

## Discussion

There is a great need for a field camera to reliably capture images of small animals [18]. The amount of work that researchers have put into making PIR systems work for small animals has been extensive [19, 21, 27, 30]. There has been greater success in using PIR cameras for capturing images of small mammals [27], [30] and our results support this. However, our experiments confirm that even at the most sensitive settings, passive infrared cameras have low probabilities of detecting many species, particularly those that are slow moving or do not have large temperature differentials with the surrounding environment. This would encompass most reptiles, amphibians, large invertebrates, and small mammals in warmer environments. We have shown that the HALT has a near perfect detection probability for all small animals regardless of size or body temperature, and that it performs well under different field conditions. This system will allow researchers to conduct passive and reliable sampling for studies designed to inventory and monitor wildlife communities, estimate occupancy and abundance, and to monitor animal behavior on a vast array of small mammal, amphibian, reptile, and large invertebrate species.

The HALT system saves time, cost, and potential harm to small animals from live-trapping. Live-trapping methods are labor intensive, require specialized permits for handling sensitive species, and can cause animals stress and injury [22–26, 28, 29]. The cost savings for using passive camera traps is also well documented [27]. In addition, managing digital camera traps to monitor wildlife does not require permits.

The HALT system is applicable to small animal studies designed to detect species presence, spatial occupancy, relative activity, habitat selection, and road underpass use. Mark recapture methods are often necessary to estimate abundance or document reproductive characteristics of study species [36]. Therefore, this methodology is not meant to replace all sampling needs for small animal studies. However, identification of individuals by spot patterns, particularly with image-recognition software has been shown to be successful for many amphibian and reptile species [37–40]. In addition, the HALT threshold can accommodate pit-tag readers, increasing its potential for use in mark-recapture studies [41].

It is necessary for cameras targeting small animals to be set with a shorter focal length and closer distance (within 1.5m depending upon size of subject) to achieve sufficient picture resolution to identify animals to species. Both PIR and HALT systems were not able to capture images of targets at high speed (1 m/sec) due to the trigger speed limitations inherent in each camera, relative to the field of view. We have observed that deer mice frequently stop and slow down when encountering the threshold as observed on video. This may help to increase the detection probability of some faster moving animals. The HALT system also has the advantage of capturing images of slow moving animals that do not trigger PIR camera systems opening up new possibilities for studies of large invertebrates and slower vertebrates, such as small salamanders.

Although the HALT system reduces false triggers in comparison to PIR activated systems, the camera may be triggered by objects that intercept the optical beam, such as leaves, small insects, or water droplets. Although we experienced some false triggers from insects crawling inside the 3 mm extension tubes during our field studies, this has since been solved by redesign. The threshold has been designed to deflect most falling objects; however, we recommend a cover be placed over the system in areas that experience frequent rain or falling debris. Drift fences are commonly used to increase capture rates of small animals for pitfall trapping (22–29). In order to maximize detections, we recommend the threshold be placed perpendicular to a drift fence or wall in order to guide or funnel animals to the optical trigger. Finally, bait can be placed on either side of the threshold if needed to attract target species. Currently, the HALT requires approximately 0.5 Ah/24 hour period so that a small rechargeable lead-acid battery (5–8 Ah) can run the system continuously for one to two weeks. This is a reasonable time period to inspect the camera and HALT threshold, add bait (if used), and to swap out the SD card and battery.

## Conclusion

The HALT system is a novel active camera trap system that is useful for sampling small animals, particularly reptiles, amphibians, small mammals and large invertebrates. It surpasses the detection ability of commonly used PIR detectors for this application and eliminates many problems associated with highly sensitive PIR camera traps, such as multitudes of false triggers and high variability in detection rates among cameras and study locations. It also surpasses previously used active trigger systems, such as the Trailmaster 1500 and 1550, by using a smaller pre-aligned beam fixed in height across a solid threshold to capture small animals. The threshold is designed to divert falling leaves and detritus. This system is designed as a niche method for detecting small animals traversing constricted pathways such as small tunnels, narrow trails, small clearings and along walls or drift fencing.

The components of the HALT system have numerous patents pending and are anticipated to be available to researchers in 2018.

## Supporting information

S1 Dataset Controlled study. Trigger: PIR = Passive Infrared, HALT = Hobbs Active Light Trigger. Brand: B = Bushnell, R = Reconxy. Analog_temp: 21°C, 24°C, 35°C. Analog_size: XS, SM, MED. Analog_speed: 1cm/sec, 20cm/sec, 1.0m/sec. S1-S6: Replicates.
(XLSX)

S2 Dataset Buena Vista field trial. (XLSX)

S3 Dataset Stanford field trial. (XLSX)

S1 Appendix Field images of species (or genera) captured using HALT trigger at Buena Vista and Stanford. (PDF)
