# Supplementary material for: An improved camera trap for amphibians, reptiles, small mammals, and large invertebrates
Source: PLoS One. 2017 Oct 5;12(10):e0185026. doi: 10.1371/journal.pone.0185026 (PMC5628828; doi:10.1371/journal.pone.0185026)
Supplement: S1 Appendix — (PDF) [file pone.0185026.s004.pdf]

S1 Appendix

Field images of species (or genera) captured using HALT trigger at Buena Vista and Stanford.

Group	Species	Picture
Amphibian	California Tiger Salamander (*Ambystoma californiense*)	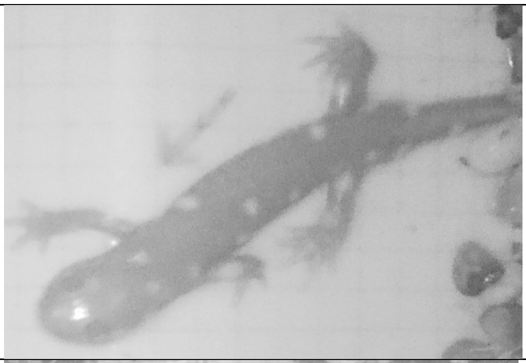
Amphibian	Santa Cruz Long-toed Salamander (*Ambystoma macrodactylum croceum*)	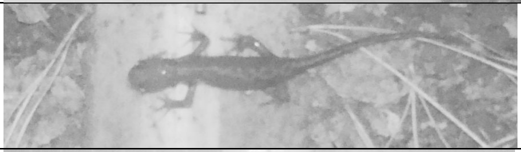
Amphibian	Western toad (*Anaxyrus boreas*)	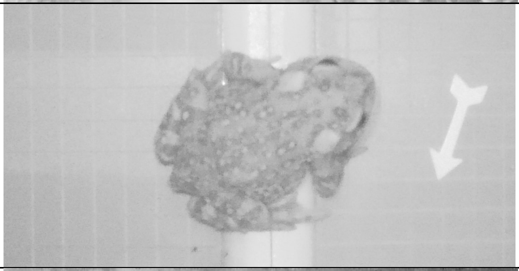
Amphibian	Arboreal salamander (*Aneides lugubris*)	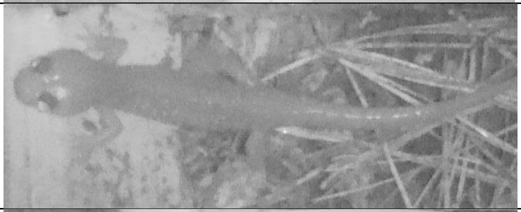
Amphibian	Slender salamander (*Batrachoseps attenuatus)*	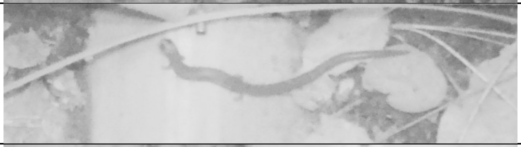
Amphibian	Ensatina salamander *(Ensatina eschscholtzii*)	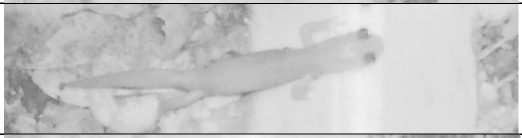
Amphibian	Pacific tree frog (*Pseudacris regilla*)	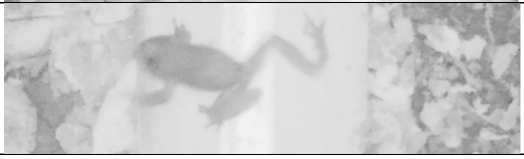

Bird	Dark-eyed junco<br>(*Junco hyemalis*)	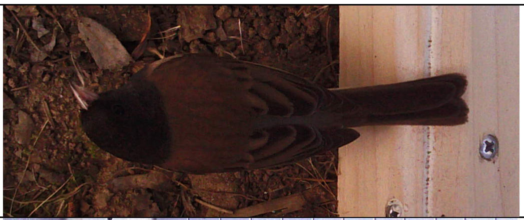
Bird	California towhee<br>(*Melozone crissalis*)	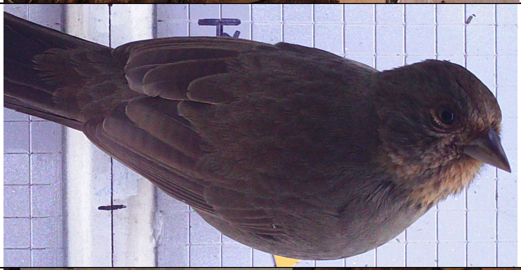
Bird	Bewick's wren<br>(*Thryomanes bewickii*)	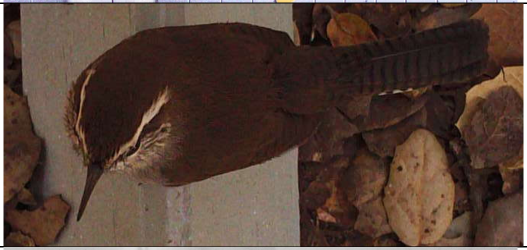
Invertebrate	Tarantula<br>(*Aphonopelma eutylenum)*	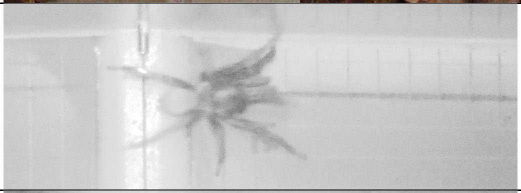
Invertebrate	Spider<br>(*Araneae*)	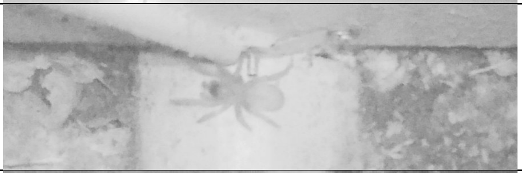
Invertebrate	Banana slug<br>(*Ariolimax)*	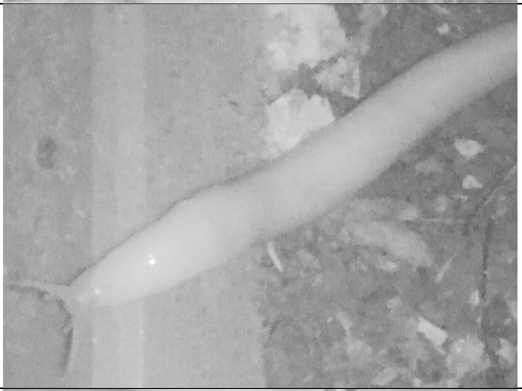
Invertebrate	Black beetle<br>(*Carabidae*)	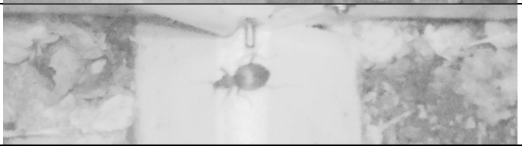

Invertebrate	Darkling beetle<br>(*Coleoptera*)	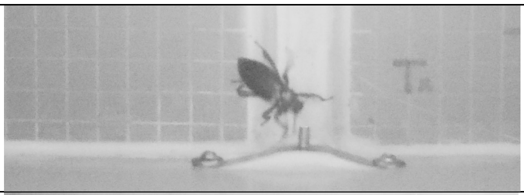
Invertebrate	Millipede<br>(*Diplopoda*)	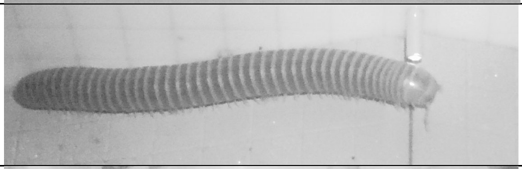
Invertebrate	Snail<br>(*Gastropoda*)	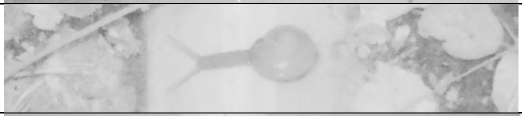
Invertebrate	Cricket<br>(*Gryllidae*)	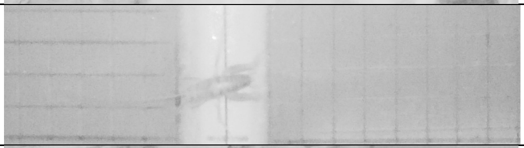
Invertebrate	Cellar spider<br>(*Pholcidae*)	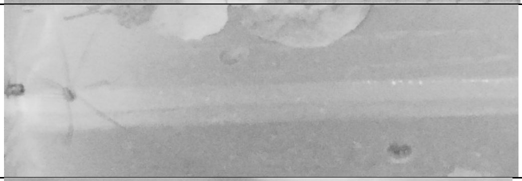
Mammal	California vole<br>(*Microtus californicus*)	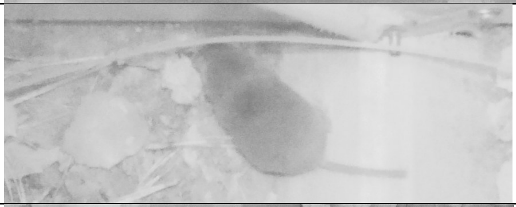
Mammal	Dusky-footed woodrat<br>(*Neotoma fuscipes)*	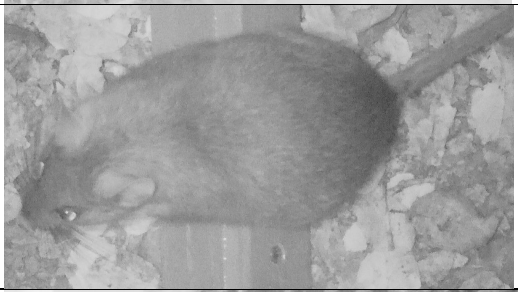
Mammal	Brush mouse<br>(*Peromyscus boylii)*	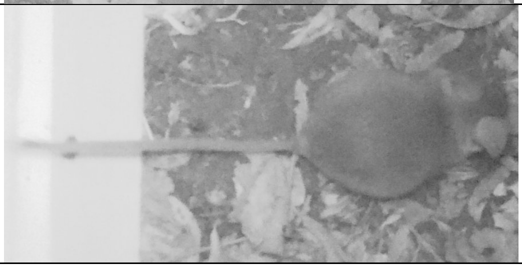

Mammal	Deer mouse (*Peromyscus maniculatus*)	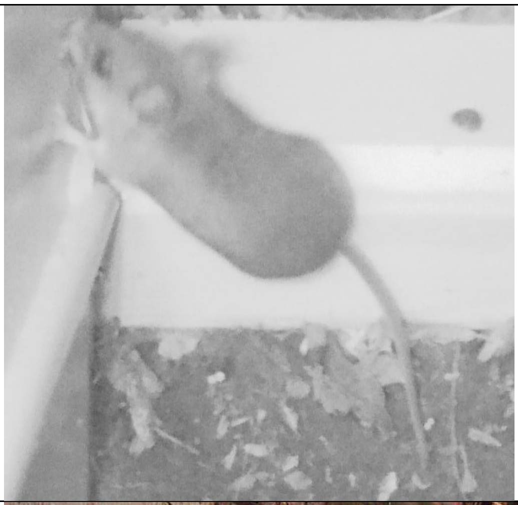
Reptile	Western fence lizard (*Sceloporus occidentalis*)	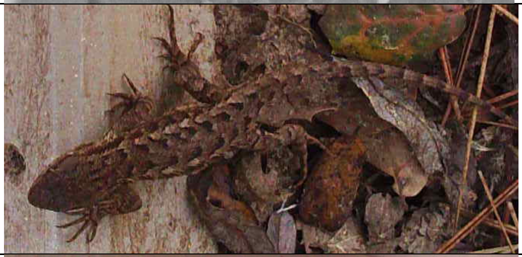
Reptile	Coast garter snake (*Thamnophis elegans terrestris)*	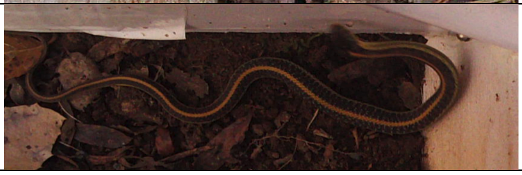
